# Supplementary material for: Recurrent circuits within medial entorhinal cortex superficial layers support grid cell firing
Source: Nat Commun. 2018 Sep 12;9:3701. doi: 10.1038/s41467-018-06104-5 (PMC6135799; doi:10.1038/s41467-018-06104-5)
Supplement: Supplementary file 1 — Supplementary Information [file 41467_2018_6104_MOESM1_ESM.pdf]

Supplementary Information

Recurrent circuits within medial entorhinal cortex superficial layers support grid cell firing

Zutshi *et al.*

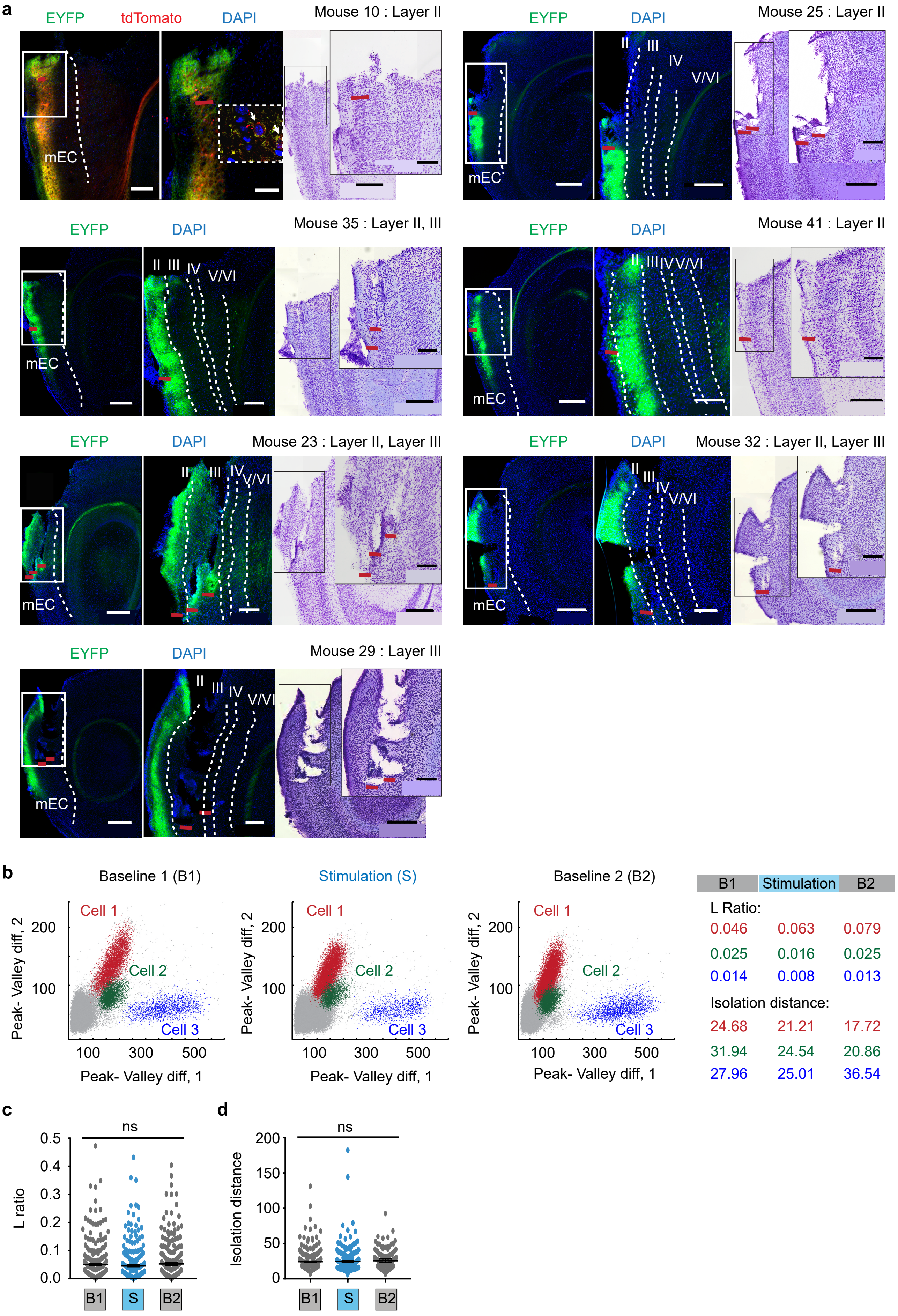
a
EYFP
tdTomato
DAPI
Mouse 10 : Layer II
mEC
Mouse 25 : Layer II
II
III
IV
V/VI
Mouse 35 : Layer II, III
Mouse 41 : Layer II
Mouse 23 : Layer II, Layer III
Mouse 32 : Layer II, Layer III
Mouse 29 : Layer III
b
Baseline 1 (B1)
Stimulation (S)
Baseline 2 (B2)
Cell 1
Cell 2
Cell 3
Peak- Valley diff, 2
Peak- Valley diff, 1
100
200
300
500
B1
Stimulation
B2
L Ratio:
0.046 0.063 0.079
0.025 0.016 0.025
0.014 0.008 0.013
Isolation distance:
24.68 21.21 17.72
31.94 24.54 20.86
27.96 25.01 36.54
c
ns
L ratio
0.0
0.1
0.2
0.3
0.4
0.5
B1
S
B2
d
ns
Isolation distance
0
50
100
150
200
B1
S
B2

Supplementary Figure 1. Histology allowed for localization of recording sites to mEC LII or LIII.

(**a**) Fluorescent and brightfield images of all 7 animals for which speed, HD, and grid cells were analyzed. The mouse number is indicated to the top right of each panel along with the mEC layers where recording sites were located. *Left of each panel*, Sagittal section of the mEC with ChR2 expression in *green*, and DAPI in *blue*. Scale bar, 500 µm. Mouse 10 is an example of a Wfs1-cre mouse crossed with an Ai14 reporter line, in which all Wfs1+ cells were labelled *red*. Overlay of *green* and *red* confirms good viral expression and spread. In all other mice, the border between LII and LIII is easily observable by EYFP expression restricted to LII. *Middle of each panel*, Magnified image of the *boxed region* from the fluorescent section on the left, with layers of the mEC outlined by *stippled lines*. *Red bars* indicate the end of tetrode tracks. Scale bar, 200 µm. *Right of each panel*, The same section as used for the fluorescent image was stained with cresyl violet and imaged using a brightfield microscope. Scale bar, 500 µm. While the deeper damage along tracks is from advancing tetrodes and the optical fiber, the tears in the tissue were observed to be caused by the postmortem extraction of the implant. Shown are magnified images of the *boxed area*, with tetrode tracks highlighted by *red bars*. Scale bar, 200 µm. Based on histological confirmation of tetrode location and the continuous monitoring of tetrode history and depth, almost all cells could be unambiguously localized to either LII or LIII. (**b**) Examples of cluster isolation and stability. Scatterplots of the spike amplitude (peak-to-valley) on two channels of a tetrode are plotted for three recording sessions – baseline 1, stimulation, and baseline 2. Clusters of three different cells are shown, with their L ratio and isolation distance for each of the sessions provided on the right. (**c, d**) We ensured that cluster quality was not compromised during optical stimulation sessions by calculating L-ratio and isolation distance which are presented as mean ± SEM of all mEC cells (n = 278 cells from 7 mice) that were included in the analysis. For both metrics, there was no difference in the cluster quality between the three recording sessions (L-Ratio: Kruskal-Wallis test, H(3) = 3.591, $p = 0.166$; Isolation distance: Kruskal-Wallis test, H(3) = 1.692, $p = 0.4292$).

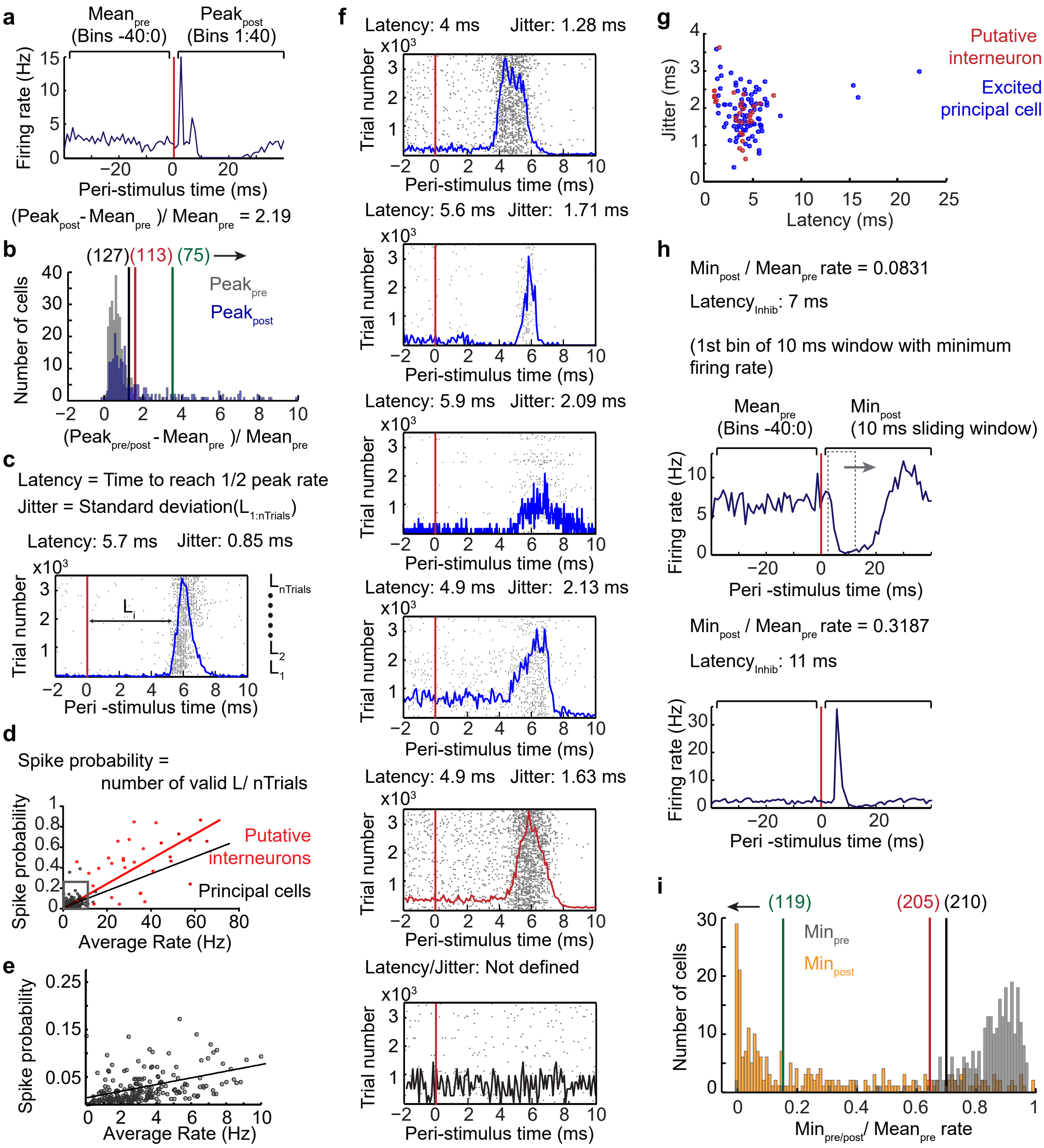

Supplementary Figure 2. Description of assignment to optogenetic response categories.

(**a**) Peri-stimulus time histogram (PSTH, 1 ms bins for ± 40 ms before and after light onset) of an example cell. The PSTH shows a fast excitatory response followed by a longer inhibitory response. The baseline firing rate of the cell ($Mean_{pre}$) was defined as the average firing rate between 40 and 0 ms before light onset. The relative increase in firing rate was then determined for the bin with the highest firing rate ($Peak_{post}$, 2.19 for the example) between 1 and 40 ms after light onset. (**b**) The increase in firing rate that is observed by chance was quantified by determining the maximum relative increase in firing rate during baseline (between 40 and 0 ms before light onset) for each cell ($Peak_{pre}$, *grey bars*). The 90th, 95th and 99th percentiles of the baseline distribution over all cells are shown by the *black*, *red*, and *green* vertical lines respectively, and the numbers above each line correspond to the number of cells for which $Peak_{post}$ (*blue bars*) exceeded each of these thresholds. For all further analyses that classify a cell as excited, we used the 95th percentile threshold. For visualization, the x-axis was limited to a 10-fold increase from baseline, which excluded 36 cells that were excited more than 10-fold from the display but not the analysis. (**c**) We examined whether cells could be classified as directly or synaptically excited by generating a peri-stimulus time raster for every light trial using 0.1 ms wide time bins extending to 10 ms after light onset. In addition, the PSTH was calculated and is shown as a *line* within the plot for each cell. The latency for excitation of each cell was defined as the time to reach half of the peak firing rate. For calculating the jitter, the time until the first spike after light onset (L_i) was first determined and the standard deviation of single-trial times to spike was then calculated. For the example provided here, the latency was 5.7 ms while the jitter was 0.85 ms. (**d**) A third measure commonly used to distinguish direct versus synaptic excitation – the probability (or reliability)

to spike in response to each light pulse was also calculated (i.e., the proportion of light pulses that were followed by at least one spike within 10 ms after light). However, this measure proved to be biased because the spike probability was strongly correlated with the baseline firing rate of a cell. Scatter plot of spike probability versus firing rate for principal cells (*black*) and interneurons (*red*) with solid regression lines indicating a correlation for both cell types (Spearman's rank correlation, n = 236 from 7 mice, R = 0.5117, $p < 0.0001$ and Pearson's correlation, n = 30 from 7 mice, R = 0.4278, $p = 0.0184$, respectively). (**e**) The same plot as in (d) but magnified for the data within the *black box*. A clear relationship between spike probability and firing rate was confirmed even for lower firing rate cells. (**f**) Examples of excited cells (PSTHs in *blue*), an interneuron (PSTH in *red*) and a non-responsive cell (PSTH in *black*), and their respective latencies and jitters. (**g**) There was no clear boundary in the latency and jitter to allow for a distinction between directly and indirectly excited cells. Therefore, cells were not classified as either directly or indirectly excited. Also shown are interneurons (*red*) that were indirectly excited after LIIP cell activation. The jitter and latency of these cells are interspersed with those of excited principal cells. (**h**) Inhibition was calculated by defining $Mean_{pre}$ as in (a) and by then defining Min_{post} as the lowest firing rate of any 10 ms sliding window between 1 and 40 ms after light onset. The onset of inhibition was the first bin of the 10 ms window with the minimum firing rate. Shown are two examples, one with a purely inhibitory response and one with an excitatory-inhibitory response. (**i**) The minimum firing rate during baseline (-40 ms to 0 ms) was identified and divided by the mean at baseline ($Min_{pre}/Mean_{pre}$) for each cell (*grey bars*). The 90th, 95th and 99th percentiles of the baseline distribution over all cells are shown by the *black*, *red*, and *green* vertical lines respectively, and the numbers above each line correspond to the number of cells for which the minimum between 0 and 40 ms after light onset (Min_{post}/ $Mean_{pre}$, *yellow bars*) was below these thresholds. For all further analyses that classify a cell as inhibited, we used the 95th percentile threshold.

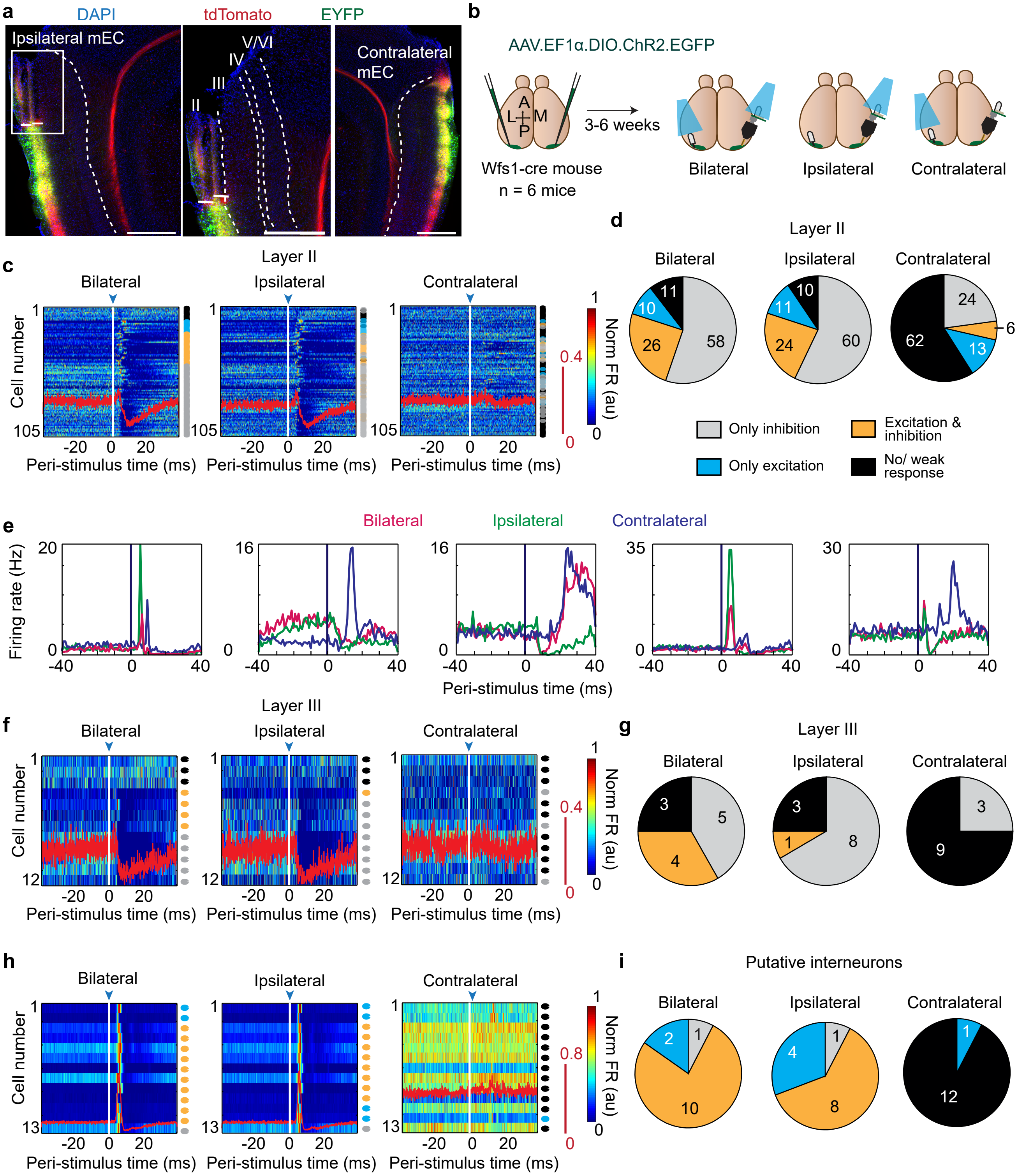

Supplementary Figure 3. Comparisons between responses to bilateral, ipsilateral and contralateral stimulation revealed only minor effects of the contralateral projections.

(**a**) Sagittal sections of a representative Wfs1-CreER x Ai14 mouse with ChR2 expression (*green*) in both hemispheres. Wfs1+ cells are labeled in *red*. *Middle*, Magnified image of the *boxed region* in the image to the left highlights where single unit recordings were performed. White lines indicate the end of tetrode tracks and confirm that recording locations were restricted to LII in this mouse. Across all mice with bilateral expression (n = 6), cells were identified as recorded from either LII or LIII. Scale bar, 500 µm. (**b**) Schematic depicts experimental design. Mice were bilaterally injected with a Cre-dependent AAV expressing ChR2-EGFP and implanted with an optic fiber cannula over the left hemisphere and an optetrode over the right. While recording from the same set of mEC cells, stimulation was performed bilaterally as well as ipsilaterally and contralaterally from the recording site. (**c**) Heat maps for mEC LII cells, sorted by excitatory versus inhibitory responses. Each row corresponds to a single cell that is normalized to its peak firing rate. The sorting was performed based on the bilateral condition, and was retained for ipsilateral and contralateral stimulation sessions. The ipsilateral

stimulation responses of individual cells were almost identical to the bilateral stimulation. In response to only contralateral stimulation, the cells often did not respond but occasionally showed late latency responses. The colored circles on the right indicate the response category of each cell, according to the legend in (d). (**d**) The 105 LII cells shown in (c) were assigned to four different categories by their responses to bilateral, ipsilateral and contralateral light stimulation. Stimulation of Wfs1+ cells in the contralateral hemisphere only led to weak and longer latency responses in a few LII cells, consistent with results from viral tracing that showed only sparse projections to the contralateral hemisphere (**Fig. 1**). (**e**) Examples of 5 cells that showed the largest amplitude responses to contralateral stimulation, with their bilateral and ipsilateral responses overlaid. In the few cells that responded to contralateral stimulation, the responses were much slower than for ipsilateral stimulation. (**f**) The same graphs as (c), but for cells recorded in mEC LIII. LIII cells were mostly inhibited by bilateral and ipsilateral stimulation. No responses were observed following contralateral stimulation except for 3 cells which responded by weak inhibition. The colored circles on the right indicate the response category of each cell, according to the legend in (d). (**g**) Quantification of responses of LIII cells in (f) following bilateral, ipsilateral and contralateral light stimulation. Color code as in (d). (**h**) Reponses of putative interneurons (classified as in **Fig. 2f**) to bilateral, ipsilateral, and contralateral stimulation. Strong excitation was only observed for stimulation ipsilateral to the recording site. The *colored circles to the right* indicate the response category of each cell, according to the legend in (d). (**i**) Quantification of the responses depicted in (h). Color code as in (d).

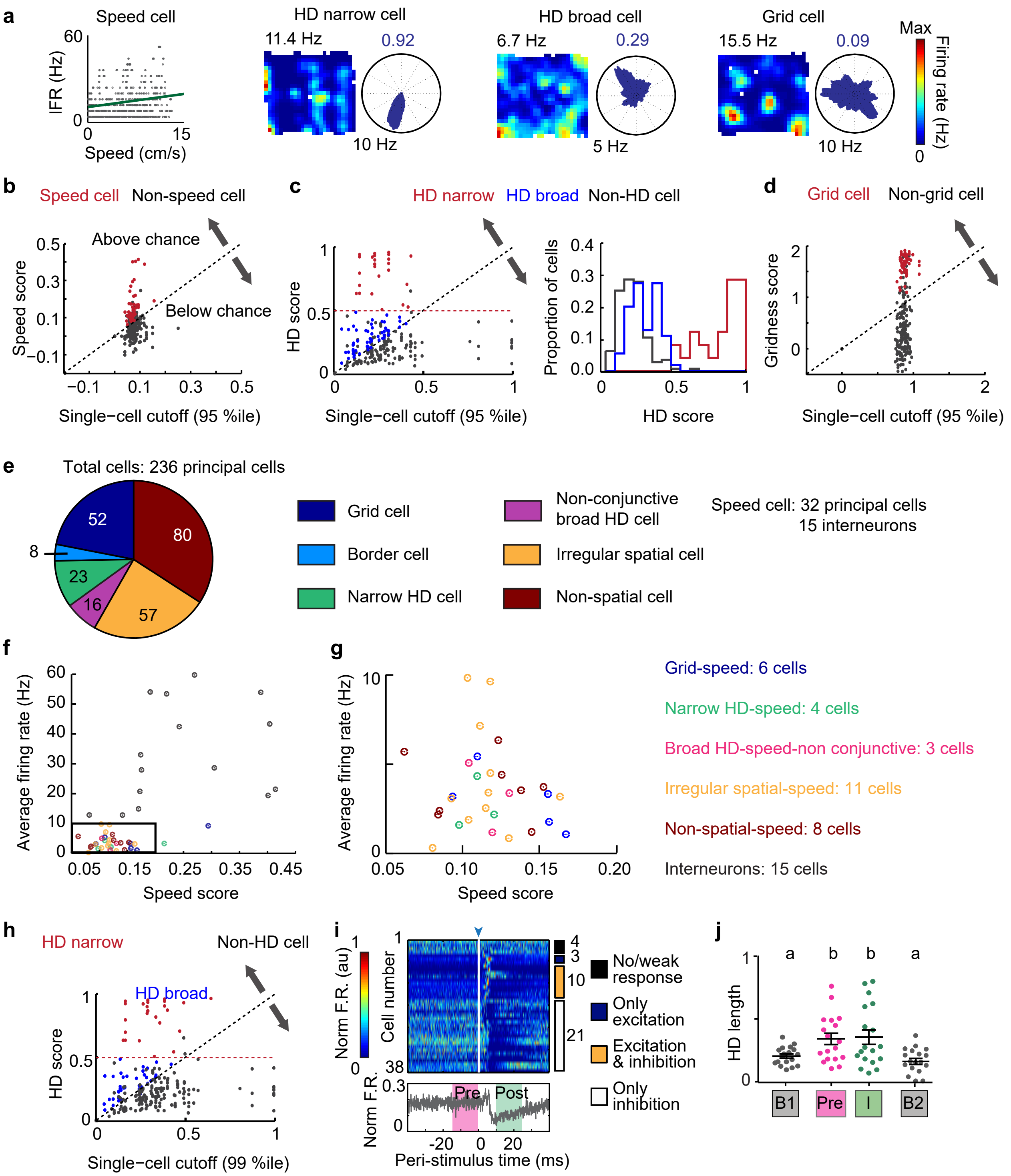

Supplementary Figure 4. Classification of cells into speed, HD narrow, HD broad, and grid categories.

(**a**) Representative examples of a speed cell, a HD narrow cell, a HD broad cell, and a grid cell. Rate maps are shown with the color scale to the right. The peak rate of rate maps is listed above each plot, and the peak rate of the polar plots is provided below each plot. The HD score is provided above each polar plot in *blue*. (**b, c, d**) Scatter plots of the maximum speed, HD and gridness scores of all LII and LIII cells across all sessions versus the 95th percentile of scores from shuffled data. The stippled line indicates the cutoff, and any cell above the cutoff was classified as positive for that score (*red*). For a cell to be considered positive for a certain category, it was required to be above the cutoff in at least 2 out of the 3 sessions (baseline 1, stimulation, and baseline 2). Thus, *grey* dots above the line are cells that were above the threshold in only one session. (**b**) Speed cells have a continuous distribution, yet there are also some extreme examples of speed tuning. (**c**) *Left,* HD cells could clearly be divided into two separate categories – HD narrow cells, those with a HD score > 0.5 (*red*), and HD broad cells, those that had a HD score above the cutoff but below 0.5 (*blue*). *Right,* Histogram of the HD scores divided by category demonstrate the bi-modality in HD scores. (**d**) Grid cells formed a distinct sub-population from non-grid

cells. (**e**) Distribution of grid, border, HD, irregular spatial and non-spatial cells across all 236 principal cells. Any conjunctive HD x spatial cells were counted in their respective spatial category, and not in the broad HD cell category. (**f**) Distribution of the speed score versus average firing rate for all speed cells. 15 out of 47 speed cells were interneurons, whereas most of the remaining principal cells had conjunctive properties. (**g**) Magnification of the *black boxed* region in (f). The conjunctive properties of all speed cells are highlighted according to the color code in (e). (**h**) Scatter plots of the maximum HD score of all LII and LIII cells versus the 99-percentile of scores from shuffled data. All narrowly tuned head direction cells that were originally classified using a 95th percentile cutoff were also above the 99-percentile threshold. Of the broad HD cells (*blue dots*), fewer (38 rather than 58) were selected with the more stringent threshold. (**i**) Peri-stimulus time plot for all broad HD cells that were selected with the 99-percentile cutoff. The overall responses of the cells are similar to those observed with a 95-percentile cutoff (see **Figure 6b**). (**j**) Quantification of HD tuning of all inhibited broad HD cells (classified using a 99-percentile cutoff) shows similar results as those observed with a 95-percentile cutoff (One-way repeated measures ANOVA followed by Tukey's multiple comparisons test, n = 18 cells from 7 mice, F (1.515, 25.76) = 9.958, $p = 0.0014$). Significant differences between conditions ($p < 0.05$) are denoted by different letters (a, b). All summary plots show the mean ± sem.

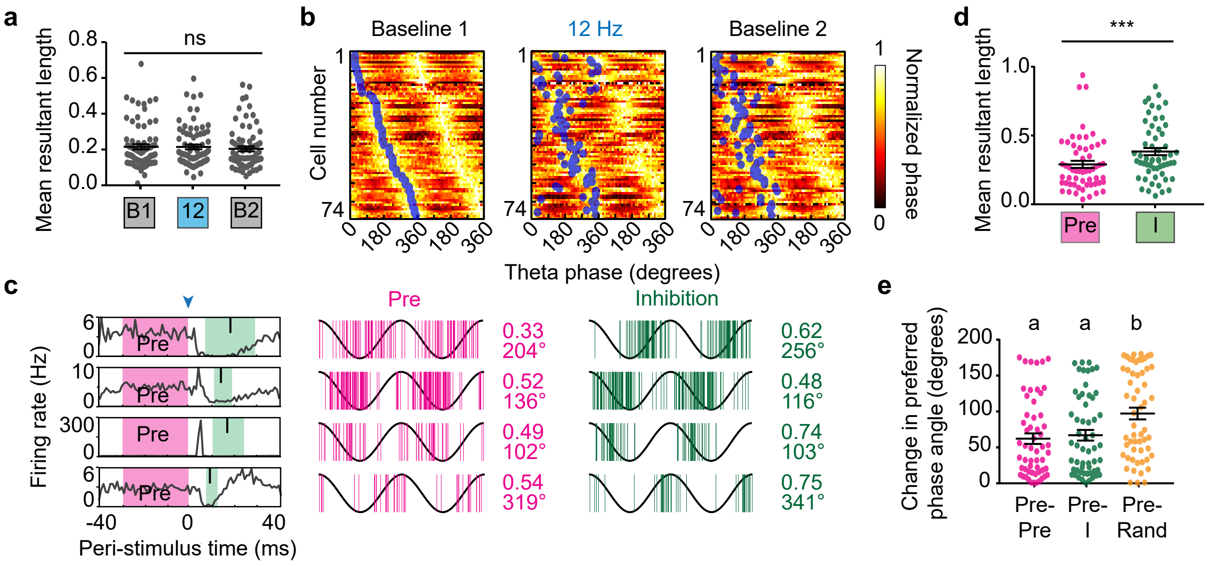

Supplementary Figure 5. Theta modulation and theta phase preference were preserved during inhibition.

(**a**) The mean resultant length for spike phase preference of theta-modulated cells (i.e., cells with mean resultant length > 0.1) was unaffected during 12 Hz stimulation sessions compared to baseline (Friedman test, n = 74 cells from 7 mice, FM(3) = 4.649, p = 0.0978). (**b**) The spike phase distribution of theta modulated cells was examined by ordering cells by their preferred phase angle during the first baseline. Each row corresponds to a single cell and blue circles represent each cell's peak theta phase angle. The order of cells was maintained across sessions. (**c**) Shown are 4 examples of inhibited theta modulated cells, where spikes during inhibition (until each cell recovered to 50% of its firing rate; I, *green*) were selected. An equal number of spikes were randomly selected from the period between 30 and 0 ms before light onset (Pre, *magenta*). The phases of each of the sets of spikes are plotted with reference to two example theta cycles, and the mean resultant length and angle for each cell is provided for Pre (*magenta font*) and I (*green font*). (**d**) Enhancement of theta modulation for spikes during inhibition (spike phase length: Wilcoxon matched-pairs signed rank test, n = 57 cells from 7 mice, W(57) = 815, $p = 0.001$). (**e**) The absolute difference in the preferred phase angle was estimated between I versus Pre spikes and, for comparison, between two sets of randomly drawn Pre spikes. The phase difference that could be reached if the phase difference were at chance levels was calculated by comparing Pre versus Pre spikes shuffled for cell identity. While there was some variability in the preferred phase angle between I and Pre, there was no difference to the Pre-Pre controls. For both conditions, the phase angle was more consistent than chance (Friedman test followed by Dunn's multiple comparisons test, n = 56 cells from 7 mice, FM(3) = 12.49, $p = 0.0019$). Significant differences between conditions ($p < 0.05$) are denoted by different letters (a, b). All summary plots show the mean ± sem. *** $p < 0.001$.

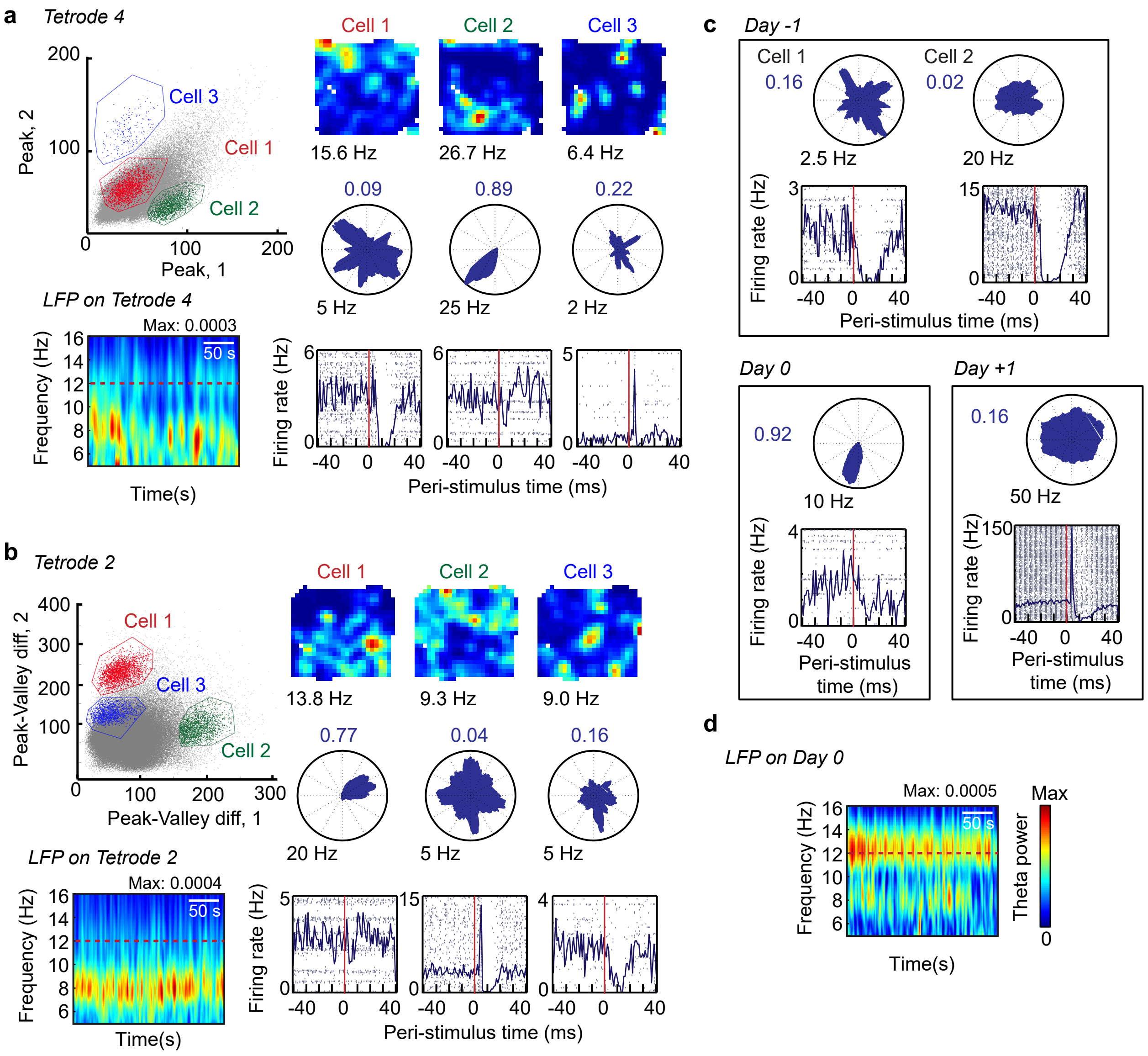

Supplementary Figure 6. Narrowly tuned head direction cells were recorded simultaneously with other cells that responded to the stimulation.

(**a, b**) Examples of tetrodes with three simultaneously recorded cells, one of them being a narrow HD cell. *Top-left of each panel,* Scatterplots of the spike amplitude (peak or peak-to-valley) on two channels of a tetrode, where clusters of the three different cells are shown. *Bottom-left of each panel*, Spectrogram of the LFP recorded on the same tetrode during the stimulation session, with power in the 12 Hz band arising due to population spikes from LIIP activation. *Top-right of each panel*, Rate maps and HD polar plots, with the peak rate indicated below the plot for each of the three cells. Rate maps are color coded from blue (minimum firing rate) to red (maximum firing rate). *Bottom-right of each panel*, PSTH of the corresponding cell during the stimulation session. While the narrowly tuned HD cells showed weak to no responses, other simultaneously recorded cells were robustly activated or inhibited, suggesting that the weak response in narrow HD cells is unrelated to ChR2 expression. (**c**) Several narrow HD cells were recorded on tetrodes without simultaneously recorded cells. In such cases, we confirmed robust responses to ChR2 stimulation by examining cells that were recorded on the same tetrode on days before and after the narrow HD cell recordings. This analysis confirmed that weak responses of narrow HD cells cannot be explained by weak ChR2 expression. In the example shown here, the HD cell was recorded on day 0. Two cells that were recorded on the day before were strongly inhibited, and one interneuron that was recorded on the day after was strongly excited and inhibited. (**d**) Spectrogram of the LFP recorded on day 0 from the same tetrode and stimulation session as (c), showing large population spiking activity at 12 Hz, suggesting that the optogenetic stimulation was effective during the session.

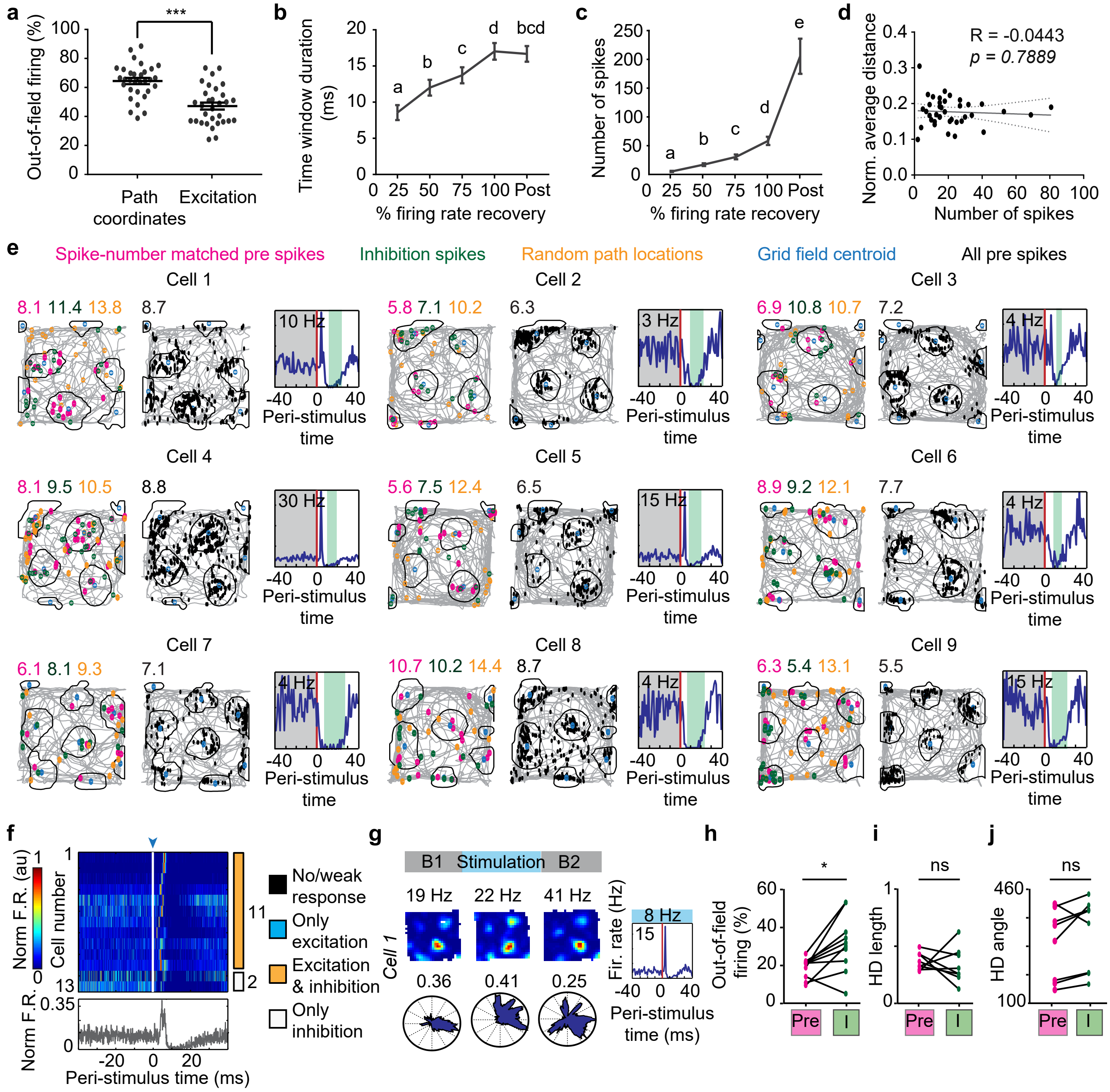

Supplementary Figure 7. Grid cell accuracy was transiently reduced during inhibition.

(**a**) Spikes that occurred during excitation (between 0 and 10 ms after light onset) exhibited substantially increased out-of-field firing (**Fig. 7d, e**), but this value remained below chance levels, which was calculated by comparison with randomly selected path coordinates (Paired samples t-test, n = 31 cells from 3 mice, t(30) = 6.676, *p < 0.0001).* (**b**) The time window duration until 25%, 50%, 75% and 100% of the baseline firing rate was regained (**Fig. 8e**). Post refers to the window between 100% firing rate recovery and 40 ms after light onset. One-way repeated-measures ANOVA followed by Tukey's multiple comparisons test, n = 46 cells from 3 mice, F (1.286, 57.87) = 15.27, *p < 0.0001.* Significant differences between conditions (*p < 0.05*) are denoted by different letters (a, b, c, d). (**c**) The total number of spikes that occurred during time windows until 25%, 50%, 75% and 100% of the baseline firing rate was reached (I) and during Post. One-way repeated-measures ANOVA followed by Tukey's multiple comparisons test, n = 50 cells from 3 mice, F (1.069, 52.36) = 39.7, *p < 0.0001.* Significant differences between conditions (*p < 0.05*) are denoted by different letters (a, b, c, d, e). (**d**) Scatter plot of the number of spikes that were included in the 50% inhibition time window, and the normalized average distance of those spikes from the grid node center for each cell. The two values were not correlated, suggesting that spike number did not affect the distance from defined grid nodes (Spearman's rank correlation, n = 39 cells from 3 mice, R = -0.0443, *p =0.7889*). (**e**) Eight examples of inhibited grid cells that became less accurate during inhibition, and one (Cell 9) that appeared to remain intact. *Left of each panel*, Path of the animal in *grey* with the grid field boundaries drawn in *black* and the centroid of each field denoted by a *blue dot*. The location of each spike that occurred during the inhibition window is denoted by a *green dot*. The location of an equal number of randomly extracted spikes from the period between 30 ms and 0 ms before light onset are shown as *magenta dots*. In *yellow*, the location of an equal number of randomly

selected points along the path of the animal are represented. The average distance of each set of locations from the grid field centroid is provided above each trajectory map. *Middle of each panel*, Path trajectories and grid field boundaries, with the location of all spikes between 40 and 0 ms before light onset in *black*. All Pre spikes were a sub-sample of these baseline spikes. *Right of each panel*, PSTH of the same cell with the inhibition window indicated in *green* and the baseline window in *grey*. Light onset is marked by the *vertical red line*. (**f**) Peri-stimulus time raster for all grid conjunctive HD cells, ordered by optogenetic response category. The number of cells in each response category is indicated to the right. Most grid by conjunctive HD cells showed excitatory-inhibitory responses, as also seen in the mean normalized firing rate. (**g**) An example grid x HD conjunctive cell and its response to optogenetic light stimulation. Rate maps are color coded from blue (minimum firing rate) to red (maximum firing rate, provided above each plot). (**h**) The out-of-field firing percentage of all grid x HD conjunctive cells was increased during inhibition compared to baseline (Paired samples t-test, n = 10 cells from 3 mice, t(9) = 3.175, $p = 0.0113$). 3 cells were excluded because they did not have any spikes during 50% recovery of their baseline firing rate. (**i**) The accuracy of HD tuning of all grid x HD conjunctive cells was not impaired during inhibition (Paired samples t-test, n = 8 cells from 3 mice, t(7) = 0.4879, $p = 0.6405$). As in **Fig. 6**, only cells with a Pre HD tuning above their respective shuffled cutoffs were included for the analysis. (**j**) The preferred HD tuning angle of all grid x HD conjunctive cells remained comparable during inhibition (Wilcoxon matched-pairs signed rank test, n = 8 cells from 3 mice, W(8) = 22, $p = 0.1484$). All summary plots show the mean ± sem. * $p < 0.05$, *** $p < 0.001$.

Animal ID	Layer	Only inhibited	Excited and inhibited	Only excited	No response	Total number of principal cells
Mouse 10	II	4	7	9	2	22
	III	NA	NA	NA	NA	0
Mouse 23	II	1	1	0	4	6
	III	7	2	1	2	12
Mouse 25	II	20	19	4	0	43
	III	NA	NA	NA	NA	0
Mouse 29	II	NA	NA	NA	NA	0
	III	14	1	0	1	16
Mouse 32	II	8	3	0	0	11
	III	21	2	0	4	27
Mouse 35	II	11	1	4	19	35
	III	2	0	1	1	4
Mouse 41	II	24	31	4	1	60
	III	NA	NA	NA	NA	0
Total	**II**	**68**	**62**	**21**	**26**	**177**
	**III**	**44**	**5**	**2**	**8**	**59**

Supplementary Table 1. Distribution of recorded LII and LIII cells across all animals.

The total number of well-isolated LII and LIII mEC cells recorded from each animal and the distribution of responses of those cells to stimulation for each animal.

Cell number	HD score			% Reduction in firing rate from baseline	Simultaneously recorded cell?	% Reduction in firing rate of simultaneously recorded cell	Distance from previously recorded cell	% Reduction in firing rate of previously recorded cell	Distance from next recorded cell	% Reduction in firing rate of next recorded cell
	B1	Stim	B2							
1	0.2554	0.5265	0.229	98.71	No	NA	~90 um	25.01	~75 um	64.6
2	0.3921	0.3882	0.543	71.97	Yes	34.6	No turns	42.68	No cells recorded	NA
3	0.6344	0.3477	0.4862	69.23	Yes	26.65	~50 um	50.76	~50 um	51.45
4	0.5406	0.6313	0.4415	45.82	Yes	90.53	No cells recorded	NA	No cells recorded	NA
5	0.6983	0.5704	0.5423	36.75	Yes	94.68	No cells recorded	NA	~ 100 um	17.43
6	0.8039	0.8317	0.8737	36.03	No	NA	~50 um	75.47	No cells recorded	NA
7	0.9005	0.8808	0.8855	33.33	No	NA	~50 um	96.28	~100 um	77.22
8	0.9085	0.9359	0.9363	28.62	Yes	17.04	No cells recorded	NA	~50 um	71.97
9	0.8853	0.9256	Not recorded	28.42	Yes	96.28	No turns	97.5	~50 um	33.33
10	0.7243	0.7723	0.6138	27.54	No	NA	~100 um	88.65	No cells recorded	NA
11	0.9444	0.9283	0.9339	23.94	Yes	71.97	~50 um	28.62	No turns	77.22
12	0.8308	0.9065	0.8803	21.74	No	NA	~50 um	17.43	No turns	99.47
13	0.7933	0.7956	0.7594	20.22	No	NA	~50 um	65.75	~50 um	22.46
14	0.9544	0.9525	0.9605	18.65	No	NA	~150 um	73.15	No cells recorded	NA
15	0.861	0.886	0.9325	17.96	Yes	97.5	~100 um	74.38	No turns	96.28
16	0.6354	0.6084	Not recorded	14.11	Yes	96.28	No turns	97.5	~50 um	33.33
17	0.7067	0.5941	0.4043	13.43	No	NA	~50 um	90.7	No turns	75.84
18	0.9522	0.935	0.9513	13.04	No	NA	No turns	24.29	No cells recorded	NA
19	0.9434	0.9361	0.9311	7.91	Yes	36.75	No turns	93.92	No cells recorded	NA
20	0.7735	0.8119	0.8197	5.21	No	NA	No cells recorded	NA	~100 um	62.27
21	0.8475	0.817	0.7692	5.01	No	NA	~50 um	89.74	~100 um	100
22	0.8405	0.8662	0.8401	4.79	Yes	17.04	No cells recorded	NA	~50 um	71.97
23	0.8834	0.7719	0.9226	1.29	No	NA	~250 um	73.25	No cells recorded	NA

Supplementary Table 2. Responses of cells recorded from tetrodes before and after narrow HD cells were recorded.

All narrow HD cells were ordered by their inhibitory responses, and the inhibitory responses of any cells recorded on the same tetrode as the narrow HD cell are provided. 'No turns' refers to subsequent/ previous recording days when cells changed due to tetrode drift instead of manual movement of the tetrodes. *Red highlighted boxes* are responses that were classified as inhibited. *Yellow highlighted boxes* are tetrodes where no cells before or after the narrow HD cell were recorded. *Grey highlighted boxes* correspond to HD scores below 0.5. Note that the most inhibited narrow HD cells (Cells 1-3) were also those that did not have consistent HD scores >0.5, suggesting that these cells are less narrowly tuned to HD.

SUPPLEMENTARY DISCUSSION

The finding that HD tuning, theta oscillations and theta modulation of cells were unaffected by our manipulation and that tuning of speed cells was only decreased to the extent predicted by the reduced spike numbers during the period of inhibition, suggests several hypotheses on how local computations may contribute to grid firing. First, intact HD information and intact theta oscillations were not sufficient to preserve grid cell firing patterns, which excludes the possibility that exclusive integration of these two signals could give rise to grid patterns. Instead, our results are more consistent with models that predict that either firing rates or theta modulation of speed cells are locally processed to give rise to grid cells[1–3]. LIIP cells can be considered to be an integral component of the local speed cell circuitry because LIIP cells receive rate-coded speed inputs from glutamatergic cells of the medial septum[4] and have been shown to be more speed modulated than other cell types in layer II[5,6]. Our manipulation resulted in a transient but substantial inhibition of speed cells, which reduced speed coding to the extent that could be predicted from the rate reduction. It is nonetheless feasible that running speed could no longer be accurately conveyed by the few remaining speed cell spikes and that the inaccuracy of grid firing emerged from the brief perturbation of the speed signal in LIIP cells. These findings could be perceived as inconsistent with reports that inactivation of the medial septal area disrupts grid firing while not only preserving, but even improving speed tuning in the mEC[7–9]. However, even though the septal inactivation improved mEC speed tuning on average, it is important to note that the speed tuning of each individual mEC cell changed to different extents. Thus, this manipulation did not merely result in a change in the gain of speed cells, but also scrambled the information. Both the previous and our findings are therefore consistent with the possibility that grid firing becomes inaccurate when relying on integrating speed information from local speed cells.

SUPPLEMENTARY REFERENCES

1. Hasselmo, M. E., Giocomo, L. M. & Zilli, E. A. Grid cell firing may arise from interference of theta frequency membrane potential oscillations in single neurons. *Hippocampus* **17,** 1252–1271 (2007).
2. Burgess, N., Barry, C. & O'Keefe, J. An oscillatory interference model of grid cell firing. *Hippocampus* **17,** 801–812 (2007).
3. Kropff, E., Carmichael, J. E., Moser, M.-B. & Moser, E. I. Speed cells in the medial entorhinal cortex. *Nature* **523,** 419–424 (2015).
4. Justus, D. *et al.* Glutamatergic synaptic integration of locomotion speed via septoentorhinal projections. *Nat. Neurosci.* **20,** 16–19 (2016).
5. Sun, C. *et al.* Distinct speed dependence of entorhinal island and ocean cells, including respective grid cells. *Proc. Natl. Acad. Sci. U. S. A.* **112,** 9466–71 (2015).
6. Reifenstein, E. T. *et al.* Cell-Type Specific Phase Precession in Layer II of the Medial Entorhinal Cortex. *J. Neurosci.* **36,** 2283–2288 (2016).
7. Koenig, J., Linder, A. N., Leutgeb, J. K. & Leutgeb, S. The spatial periodicity of grid cells is not sustained during reduced theta oscillations. *Science* **332,** 592–5 (2011).
8. Brandon, M. P. *et al.* Reduction of theta rhythm dissociates grid cell spatial periodicity from directional tuning. *Science* **332,** 595–9 (2011).
9. Hinman, J. R., Brandon, M. P., Climer, J. R., Chapman, G. W. & Hasselmo, M. E. Multiple Running Speed Signals in Medial Entorhinal Cortex. *Neuron* **91,** 666–679 (2016).
